# Physical Fitness, Physical Activity, Sedentary Behavior, or Diet—What Are the Correlates of Obesity in Polish School Children?

**DOI:** 10.3390/ijerph14060664

**Published:** 2017-06-20

**Authors:** Stanisław H. Czyż, Abel L. Toriola, Wojciech Starościak, Marek Lewandowski, Yvonne Paul, Adewale L. Oyeyemi

**Affiliations:** 1 Physical Activity, Sport, and Recreation Focus Area, North-West University, Potchefstroom 2520, South Africa; 2 Department of Sport Didactics, University School of Physical Education in Wroclaw, 51-612 Wroclaw, Poland; wojciech.starosciak@awf.wroc.pl; 3 Department of Sport, Rehabilitation and Dental Sciences, Tshwane University of Technology, Pretoria 0183, South Africa; ToriolaAL@tut.ac.za (A.L.T.); pauly@tut.ac.za (Y.P.); 4 Department of Pedagogy, College of Management Edukacja, 50-001 Wroclaw, Poland; lewmar69@gmail.com; 5 Department of Physiotherapy, College of Medical Sciences, University of Maiduguri, Maiduguri P.M.B 1069, Nigeria; alaoyeyemi@yahoo.com

**Keywords:** obesity, overweight, children, Polish population, physical activity

## Abstract

There is substantial evidence of rising prevalence of overweight and obesity and its co-morbidities among children in western-high income developed countries. In the European Union, the prevalence of overweight and obesity is increasing fastest among Polish children. Yet, there is paucity of evidence on the relationship of behavioral factors with body weight status of children in Poland. This study examined the association of obesity with physical fitness, physical activity, sedentary behavior and diet among Polish children. A total of 641 children (10–15 years) recruited from the Lower Silesia region of Poland participated in this cross-sectional study. Participants’ anthropometrics, physical fitness, physical activity, sedentary behavior and dietary intake were assessed. Outcome variables were weight categories (according to body mass index [BMI], waist-to-hip ratio [WHR], and percentage body fat [% BF]). The strongest negative correlation was found between VO_2_max and %BF (*r* = −0.39, *p* <0.05). Significant negative correlation was also found between VO_2_max and weight categories (*r* = −0.15). Results of the multinomial logit analysis showed that VO_2_max increased in groups of overweight, normal weight and underweight children by 13%, 26% and 19%, respectively as compared to the group of obese children. VO_2_max and weight and obesity indices were strongly correlated in both gender and age groups. Education and intervention programs to increase physical fitness (VO_2_max) through aerobic training are recommended for Physical Education teachers, parents and children in order to reduce the rate of overweight and obesity among children in the Lower Silesia region of Poland.

## 1. Introduction

A number of public health studies have indicated that obesity is a complex but rapidly growing global epidemic which affects children and adults [1,2]. Contrary to documented evidence in the last fifty years, both developed and developing nations are now experiencing increasing multidimensional trends in childhood overweight and obesity [3]. A recent estimate indicates that 43 million children (35 million in developing countries) are either overweight or obese, and 92 million are at risk of being overweight [4]. Also, an upward trend in the global estimate of childhood overweight and obesity from 4.2% in 1990 to 6.7% in 2010 has been reported, and it has been projected that the trend could reach 9.1% or ≈60 million in 2020 [4]. Recent studies have also confirmed this rising trend in the prevalence of obesity in children [5,6].

Previous research has shown that excessive body weight is the sixth most important disease risk factor worldwide [7]. The need to evaluate body fat and weight during one’s formative years is of great importance for three cogent reasons. First, it forecasts the risk of overweight and obesity during adulthood. Second, childhood obesity is a risk factor for diabetes, cardiovascular dysfunction and other co-morbidities at adulthood [8,9]. Third, because obesity is strongly associated with excess visceral fat concentration [10] and has a low to moderate correlation with skeletal muscle and hepatic lipid deposition [11,12], childhood obesity is a precursor to the development of abdominal obesity and, to some extent, ectopic fat deposition later in life [9]. Recent global trends have suggested increasing prevalence of body weight disorders and associated cardiovascular and metabolic diseases in children and adolescents [13]. A number of such studies have also been carried out in European countries, for example, France [14], Poland [15], and Spain [16]. Given the fact that precursors of obesity and overweight originate from childhood, it is important to continuously monitor the levels of these risk factors in children and adolescents so that appropriate lifestyle modifications can be recommended. Cultural, social as well as economic changes in Poland are important drivers of overweight and obesity in children [15].

According to Stańczyk et al. [17] and Niemirska et al. [18], cultural and socio-economic changes in Poland have contributed to the higher prevalence of overweight and obesity in Polish children in recent years. This assertion is contrary to the old stereotypes in Poland that see excess body weight in childhood as a sign of healthy development. Rapid westernization, urbanization, and mechanization of the Polish society could potentiate unhealthy public health consequences, including overweight and obesity in children. Thus, the necessity to prevent childhood overweight and obesity in order to produce a healthy Polish population is a public health priority.

Prevalence studies on risk factors of cardiovascular disease in children and adolescents have implications for identifying and developing country-specific appropriate intervention strategies. For instance, the OLAF Study by Kulaga et al. [19] reported striking differences in normative data for the height, weight, and the body mass index (BMI) of school-age Polish children and adolescents compared with international norms. The study also showed that 30% of Polish children had waist circumference measurements above the international cardiovascular risk threshold [20].

Studies have also shown that Polish children are the fastest growing population of obese and overweight individuals in the European Union (EU) [21,22,23]. According to the World Health Organization [21], over 22% children in Poland are overweight or obese. However, whilst a number of previous studies have examined the prevalence of obesity and overweight in Polish school children [17,18,19,20], little attention has been given to investigating the behavioral factors which contribute to the development of these deleterious health conditions among Polish children.

Research on physical activity and health is cognizant of different factors associated with overweight and obesity, e.g., diet or physical activity [24,25]. However, various behavioral factors including sedentary behavior and physical fitness play different roles in the global obesity epidemic. Thus, it is important to identify behavioral factors that are the most relevant correlates of obesity in Polish children. Highlighting such correlates of obesity among Polish children could be crucial to instituting preventive actions among stakeholders such as parents, teachers, sport instructors and health professionals. Sedentary behavior is defined as “a distinct class of behavior (e.g., sitting, watching TV, driving) characterized by little physical movement and low energy expenditure” [26] whereas, physical activity is that which meets established movement guidelines (usually reflected in achieving a threshold number of minutes of moderate to vigorous physical activity per day) [26]. Physical fitness may be defined as a set of attributes that are either health-or skill-related [27]. These factors are commonly believed to be associated with overweight and obesity. However, there are very few studies examining correlations of these aforementioned behavioral factors with overweight and obesity among Polish children. Therefore, the aim of the present study was to examine the roles of diet, physical fitness, sedentary behavior and physical activity in the prevalence of overweight and obesity among Polish school children in the Lower Silesia region. This study could provide a better perspective and understanding of behavioral factors influencing overweight and obesity in Polish children.

## 2. Materials and Method

We used a cross-sectional design to evaluate the relationships between diet, physical fitness, sedentary behavior and physical activity patterns and overweight and obesity. Due to the absence of a sampling frame, we employed a non-probability convenient sampling technique to recruit participants into the study. The school children were non-randomly sampled from 21 schools of the Lower Silesia region of Poland (Table S1).

Given that we had planned to conduct Structural Equation Modeling (SEM), we assumed that the sample size had to be larger than 460, as this was determined as the maximum range for SEM by Wolf et al. [28]. Additionally, we calculated (Statistica 13) the sample size based on the following assumptions: null hypothesis the Root Mean Square Error of Approximation (RMSEA) = 0.05; alpha = 0.05; power goal = 0.9; degrees of freedom = 20. Thus, the required sample size was estimated as 573.

### 2.1. Participants

A total of 641 children from three age groups (10, 12, 14 years old) completed the study (Table 1) between 2012 and 2014. The age here indicates the age at which children start education in September each year in the 3rd and 5th grades of primary school and 1st grade of secondary school; since the study was conducted over the whole school year, children that have been, for instance, in 3rd grade were either 10 or 11. A similar situation referred to the children in 5th grade and 1st grade. In order to shorten and simplify the group description, we will call these three groups 10- (10–11 years), 12- (12–13 years) and 14- (14–15) year-old children. The 10–11- and 12–13-year-old children were in the 3rd and 5th grades of primary school, respectively, whereas the 14–15-year-old participants were attending 1st grade of secondary school (*gymnasium*). The response rate was at an acceptable level of 57.68% [29]. Six extreme outliers (above 3.29 standard deviations from the mean age) were removed from further analysis. A total of 155 recordings were not included in the analysis due to incomplete data (e.g., the questionnaire or physical test missing).

The recruiting fliers were distributed by school teachers to the parents and children after an approval was granted from the school directors. All children in the 3rd and 4th grades of primary school and 1st grade of secondary school in the selected schools were invited to participate in the study. Only apparently healthy children (free from disease or pain) were included in the study. Children who were excused from physical education classes were excluded.

### 2.2. Ethics Statement

Permission for the subjects to participate in the study described in this manuscript was approved and granted by the Committee for Ethics of the University School of Physical Education in Wroclaw, Poland. The research was conducted according to the principles in the Declaration of Helsinki. All participants took part in the study voluntarily and could discontinue their participation at any time without repercussion. The participants and their legal guardians provided assent and written informed consent, respectively, to participate in this study.

### 2.3. Procedure

Nine trained research assistants who were post-graduate students at the University School of Physical Education in Wroclaw participated in the data collection. A technical training workshop was conducted for the research assistants to enable them to competently measure the participants’ anthropometric and fitness variables. During the training workshop, each research assistant was assigned to a specific measurement protocol at a designated work station, e.g., flexibility measurement. A team leader at each test station coordinated prescribed data collection procedures. Before data collection commenced, the pupils completed the demographic section of the data inventory as well as two questionnaires. Participants’ ages were verified against school records in order to ensure the accuracy of the information provided. Data collection, including all measurements and questionnaires, took place at the schools the children were attending.

### 2.4. Measurements, Test, and Questionnaires

We collected several different kinds of information. Anthropometric and fitness measurements were conducted. Participants self-completed two questionnaires: firstly, about dietary assessment, and secondly, regarding habitual physical activity and sedentary behaviors. The participants were provided with additional clarifications if needed. The completion of the questionnaires took approximately 25 min. The fitness test procedures took approximately 2 h. The testing procedure was completed during obligatory classes (including physical education) on two consecutive days according to the teachers’ suggestions.

#### 2.4.1. Anthropometry

We used the most popular, inexpensive and reliable measures for assessing body weight and obesity, i.e., body mass index, skinfold measurements and waist-to-hip ratio [30]. Anthropometric measurements including height, weight, skinfold thickness and girth (waist and hip) were taken using the protocol of International Society for Advancement of Kinanthropometry (ISAK) [31]. From these measurements, the following variables were derived: body mass index (BMI), waist-to-hip ratio (WHR), and percentage body fat (%BF). BMI was calculated as weight (kg)/height^2^ (m). We categorized the children into four groups based on age and height to indicate those with underweight, normal weight, overweight, and obesity. We used international cut-off points for thinness [32,33] for categorization. The cut-off points for thinness by sex for exact ages were defined to pass through BMI of 18.5 [33], 25 and 30 kg/m^2^ [31], i.e., referring to the BMI cut-off points at the age of 18 (below 18.5 underweight, between 18.5 and 25—normal weight; 25–30—overweight; over 30—obese). WHR was determined as WHR = waist girth (cm)/hip girth (cm). Percentage body fat (%BF) was estimated using the following age- and sex-specific regression equation: %BF = 1.21 × (subscapular + tricep skinfold) − 0.008 − 1.7 [34].

#### 2.4.2. Cardiorespiratory Measures

A maximal multistage 20-m shuttle run test [35] was performed to determine the children’s maximal aerobic power. In the bleep test, participants ran back and forth on a 20-m course and were required to touch the restraining lines; at the same time, a sound signal was emitted from a pre-recorded tape. The frequency of the sound signals was increased 0.5 km·h^−1^ each minute from a starting speed of 8.5 km·h^−1^. When the subject could no longer keep up the pace, the last stage number announced was used to predict maximal oxygen uptake (VO_2_max) (mL/kg/min) from the speed (km/h) corresponding to that stage (speed = 8 + 0.5 stage no.) and age (year): VO_2_max = 31.025 + 3.238 × Speed − 3.248 × Age + 0.1536 × Age × Speed [35].

#### 2.4.3. Fitness Tests

The fitness variables were measured using the American Alliance for Health, Physical Education, Recreation and Dance (AAPHERD) youth fitness assessment [36], the EuroFIT Physical Fitness Test Battery (EuroFIT) [37] and the Fitnessgram [38] test batteries as appropriate. These are widely recognized tests of physical and health-related fitness assessment in children.

Flexibility (Sit and Reach Test)

Flexibility was measured using a standard sit and reach the box. This test indicates lower back and hamstring flexibility. Participants’ sat barefoot with their knees and feet placed firmly on a floor mat. They were instructed to gradually push the indicator on the ruler forward as far as possible with hands stretched, without jerking and knees fully extended. Fingers of both hands had to reach the same distance simultaneously and any bouncing movement was disallowed. The best of three trials was recorded as each participant’s criterion score, i.e., the farthest distance (in cm) reached.

Muscular Endurance (Sit-Up Test)

Muscular endurance was assessed based on the maximum number of sit-ups performed in 60 s. In a starting position, the subjects were seated on a floor mat, back straight, keeping the hands clasped behind their neck, and the knees flexed at 90 degrees with heels and feet flat on a floor mat. They were then asked to lie down on their backs, so that the shoulders touched the floor mat and returned to the sitting position with elbows pointed out in front, touching their knees, whilst keeping their hands clasped behind their neck for the whole time. The test was terminated when participants were too tired to complete a full sit-up. The total number of correctly completed movements was recorded as the number of successfully executed sit-ups.

Muscular Strength (Push-Up Test)

Muscular strength test was assessed using the push-up test. In the starting position, a participant’s abdomen was parallel to the floor with elbows extended on both sides to support the trunk, head facing forward. Both the feet were plantar flexed balancing on the flat hard surface next to each other. During the movement, the upper body was lifted from above the floor at an angle of about 60° using the shoulder muscles supported by both arms. The chest and chin were only allowed to touch the floor slightly. The test was terminated when participants were exhausted or no longer able to perform a complete push-up. The result was recorded as the number of correctly executed push-ups per minute.

Standing Long Jump

This test measured the explosive power of leg extensors. Using a double-foot take off from a half-squat posture, the participant jumped forward as far as possible. The best of three trials was recorded in centimeters.

Grip Strength

In a standing position, a participant held a handgrip dynamometer with the hand hanging downward naturally at the side at any angle between 90° and 180°. The participant then exerted maximum strength to squeeze the spring of the dynamometer as prompted by the tester. Three separate trials on the left and right hands were recorded in kg. The mean value of the best results for the left and right hands was used for further analysis.

#### 2.4.4. Questionnaires

Participants completed two self-report questionnaires: the Previous Day Physical Activity Recall (PDPAR) [39] and the Food Frequency Questionnaire (FFQ) on dietary habits. The PDPAR was translated from English into Polish, and after re-translation to English, both versions were compared. The PDPAR was adapted and modified for Polish context. The coefficient of reliability for section A (physical activity level) of the PDPAR was: Cronbach’s alpha = 0.77, for the section b (sedentary behaviors) Cronbach’s alpha = 0.58. The dietary questionnaire was constructed as the Food Frequency Questionnaire [40]. The FFQ consisted of a list of foods and a selection of options relating to the frequency of consumption of each of the foods listed. We constructed an FFQ as it is designed to collect dietary information from large numbers of individuals (100 individuals or more) and it usually is a self-administered questionnaire [40]. The coefficient of reliability for the FFQ was: Cronbach’s alpha = 0.88.

Personal Dietary Questionnaire

Although the questionnaire may consist up to 150 foods, in general, as few as 11 food items may be sufficiently informative since the major sources of the nutrients of interest are found in relatively few food types [40]. Our personal dietary assessment was completed with the use of a questionnaire consisting of forty closed-ended items, thus making it a relatively simple instrument that could be completed in approximately 15 min.

Participants were asked to indicate how frequently they consume the food listed in the questionnaire. They could mark the number(s) of their choice with an X on the following six-point Likert scale: 6 (always), 5 (usually), 4 (often), 3 (sometimes), 2 (never), 1 (do not know).

The children indicated the food they consume from the following list of items: chips (as snacks); milk and dairy products; meat, fish; eggs; vegetables; fast food (for example, from McDonald’s, Burger King, knish, pizzas, burgers, etc.), fruits, sugar, fruit juices, soft drinks, such as Coca Cola, Pepsi Cola, Fanta, Mirinda, tonic, Sprite, Nestea, Jupik, orangeade; ice creams; chocolate; hamburger; crisps; cake; yoghurt; brown bread (wholegrain); white bread; cereals; corn flakes; sweets; maize; pizza; crackers; potatoes; rice; hot-dogs; sweet rolls; margarine on bread; butter on bread; peanuts; cheese; beef; pork; chicken; nuts; olive oil (e.g., for baking); sunflower/rape oil for baking; sausage.

Habitual Physical Activity and Sedentary Behavior Questionnaire

The aim of the questionnaire was to determine the level of physical activity (PA) participation and sedentary behavior of the children.

Participation in Physical Activities

The questionnaire consisted of three questions. In the first one, children indicated all the physical activities they participated in the previous day. They were asked to recall how often they participated in the following activities: running, play ball, walking, mixed walking-running, cycling, outdoor games and plays, climbing on the playground equipment, basketball, roller-skating, volleyball, swimming, soccer, jump rope, dancing. Children could choose one of three possible answers: “*not at all*”, “*sometimes*”, “*always (every day)*”. Children could also specify other sports; however, most of their answers indicated activities not related to physical activities, such as “computer” (4 kids), or “singing” (1 kid). Answers related to PA, e.g., “ice hockey” (2 kids), “horse riding” (3 kids), “wrestling” (3 kids), “tennis” (3), “paintball” (2 kids) and so on were too few to be included for further analysis. Given the number of other sports indicated and their popularity among children, we also confirmed that sports listed in the survey were the most popular at this age group.

Sedentary Behavior

We asked participants about their sedentary behavior. Children were asked to indicate how many hours per day they spend on the nine following activities: watching TV/video/movies on computer; using a computer or playing video games; reading books or magazines; playing board games; washing dishes or cleaning the kitchen; talking on the phone, cleaning a room/house; arts; listening and playing music. Children could indicate their choice by marking an X on the five-point scale: 1 (less than 30 min), 2 (30 min – 1 h), 3 (1–2 h), 4 (2–3 h), and 5 (more than 3 h).

As it can be noticed, we decided to include questions about washing dishes or cleaning the kitchen separately from those about cleaning a room/house. We decided to do so because, in Poland, there are still regions and places where the traditional division between so-called “women’s duties” and “men’s duties” can be observed. Therefore, some girls could have more physically demanding domestic duties than boys.

### 2.5. Data Analysis

#### 2.5.1. Data Reduction—Cluster Analysis

In order to reduce information from the questionnaires referring to *Sedentary behaviors, Participation in physical activities,* and *Personal diet*, we performed a cluster analysis (see Appendix A for details). We used Ward’s method and city-block (Manhattan) distances as a distance measure. The main objective for clustering was to reduce the number of variables for further data analysis. We also wanted to determine whether there were any discrepancies between answers. Values representing each observation in the clusters were computed as an arithmetic mean of the original values of the variables included in each cluster. Means and standard deviations were computed for all dependent measurements across sex and age categories.

The analysis of personal dietary questionnaire allowed us to recognize five clusters: Fast food, Sweets, Healthy, Meat and oil, and Every day diet. They included different types of food that were represented by the name of the cluster. In the Fast food cluster, the following food items were included: chips, fast food, pizza, hamburger, hot dogs, sweet rolls, crisps, crackers, peanuts, nuts, and sausages. The Sweets cluster consisted of sugar, soft drinks, ice cream, cakes, chocolate, and sweets. In the Healthy category, the following were included: fish, eggs, rice, brown bread cereals, and corn flakes. The Meat and oil cluster comprised pork, beef, margarine on bread, olive oil, rape/sunflower oil, and maize. The last group consisted of different kinds of food: butter, white bread, cheese, potatoes, chicken, meat, fruit juices, fruits, vegetables, yogurt, milk and dairy products. Interestingly, all of the milk and dairy products were included in this category, i.e., cheese, yogurt, milk and dairy products. Also, fruits, fruit juices, and vegetables were included. The food items that were difficult to categorize in this cluster included: white bread, butter on bread, potatoes, chicken, and meat. We speculated that these products could likely have been used for the children’s last breakfast or lunch and therefore, easily remembered, were found in one category. We called it the Every day diet.

After having clustered data from the questionnaires regarding participation in physical activities we recognized four clusters: Technical PA, Niche PA, Popular PA, and Walking/running PA. In the Technical PA cluster, three physical activities were included: swimming, basketball, and volleyball. The Niche PA cluster consisted of four activities: dancing, jump-rope, roller-skating and climbing. The four most popular activities, i.e., soccer, cycling, outdoor games and plays, and play ball were clustered in the Popular PA. The fourth cluster, Walking/running PA, included three similar activities: running, walking and mixed walking/running.

Regarding sedentary behaviors, we recognized three clusters: TV/Computer, Music, and Active. The TV/Computer cluster consisted of two variables: “watching TV/video/movies on computer” and “using a computer or playing video games”. The Music cluster consisted only of one element, i.e., listening and playing music. In the Active cluster, six different activities were included: reading books or magazines; playing board games; washing dishes or cleaning the kitchen; talking on the phone, cleaning a room/house; arts.

#### 2.5.2. Correlations and Modeling

We originally planned to perform structural equation modeling (SEM) with weight indices as dependent variables and physical fitness, physical activity, sedentary behavior, and diet as independent observed variables. However, criteria for several fit indices (including RMSEA, the Normed Fit Index—NFI, the Comparative Fit Index—CFI, the Goodness of Fit Index—GFI) were below or above accepted cut-off points. This was attributed to the low correlations between variables, and a similar trend was also observed when regressions with weight indices as dependent variables were computed. Therefore, Pearson’s product-moment correlation coefficients (r) were calculated across all variables, including physical test results (i.e., flexibility, sit-ups, standing long jump distance, push-ups, grip strength and VO_2_max), habitual personal dietary clusters (i.e., Fast Food, Sweets, Healthy, Meat and oil, Every Day Diet), participation in physical activity clusters (i.e., technical PA, niche PA, popular PA, walking/running PA), and sedentary behaviors clusters (i.e., TV/Computer, Music, Active) to explore if there were any significant relationships with the weight categories (according to BMI), WHR, or %BF.

Given that the strongest correlations were found between VO_2_max and body weight measurements, we applied a multinomial logit model and calculated the odds ratios for determining the association between VO_2_max and body weight categories.

## 3. Results

### 3.1. Descriptive Statistics

The descriptive statistics on the children’s anthropometric variables according to age, and sex categories are presented as means with SDs in Table 1, and the number of children categorized as underweight, normal weight, overweight, and obesity are presented in Table 2. Descriptive statistics of physical tests are presented in Table 3.

### 3.2. Correlations between Obesity and Physical Fitness, Physical Activity, Sedentary Behavior and Diets

We calculated correlation coefficients for all participants, for sex groups (Table 4) and for different age (Table 5). We also calculated the correlation coefficients (Pearson’s *r*) between weight categories (according to BMI) and %BF and WHR. We found that there were significant correlations between these indices (all at *p* < 0.001): between WHR and %BF *r* = 0.15; between WHR and weight categories, *r* = 0.26; and between and %BF and weight categories, *r* = 0.70.

For all participants, there was a significant negative correlation (*p* < 0.05) between all physical test results excluding flexibility, and weight categories and %BF (see Table 4). The VO_2_max and %BF indicated the strongest association (*r* = −0.39). There was no such relationship between WHR and VO_2_max (insignificant difference with *r* = 0.05). On the other hand, there was a statistically significant correlation between flexibility and WHR (*r* = 0.16). WHR was also significantly negatively correlated with types of sedentary behaviors (see Table 4), but this correlation was rather weak (oscillating around *r* ≈ 0.10). A similar situation was noticed with the correlations between WHR and types of sport activities that participants were involved in (see Table 4). All of the weight indices (i.e., WHR, %BF and weight categories according to BMI) were negatively significantly (*p* < 0.05) correlated with the cluster *Walking/running PA*, which grouped activities such as walking, running, walking and running (see Table 4 for the *r* values). Different kinds of food represented by habitual personal dietary clusters were also significantly negatively (*p* < 0.05) correlated with weight categories (*r* = 0.14), %BF (*r* = 0.11) and WHR (*r* = 0.11).

The analysis of the 10-year-old participants yielded similar results as in general analysis (Table 5). Significant correlations were found between all physical tests results except flexibility and weight categories and %BF. However, for all of these significant correlations, the one with grip strength was positive, whereas the rest were negative. Specifically, the correlations between %BF and sit-ups (*r* = −0.35), standing long jumps (*r* = −0.37) and VO_2_max (*r* = −0.34) were significant (see Table 5). The %BF index was also significantly (*p* < 0.05) correlated with *walking/running PA* (*r* = −0.17).

Surprisingly, both weight category and %BF indices were negatively correlated with *Sweets* intake (both *r* = 0.25). This correlation was statistically significant (*p* < 0.05). We found only one significant correlation between WHR and the rest of the variables. There was a negative correlation between *TV/Computer* cluster and WHR wit *r* = −0.16.

In 12-year-old boys, the physical test results were significantly correlated with weight categories and %BF; however, all tests were significantly (*p* < 0.05) correlated with only %BF (Table 5). These correlations were negative except for the grip strength tests. The most significant association was found between VO_2_max and %BF, reaching *r* = −0.44. This negative correlation was also found between VO_2_max and weight categories (*r* = −0.44). The WHR index correlated with only one physical test result, i.e., flexibility (*r* = −0.29). The PA cluster was negatively correlated with weight categories and %BF (significantly at *p* < 0.05). The sedentary behavior habit, i.e., *TV/Computer* cluster, was significantly correlated with the weight categories (*r* = −0.20; *p* < 0.05).

In 14-year-old participants, there were significant negative correlations between sit-ups, standing long jump tests and weight categories and %BF (all of them at *p* < 0.05, see Table 5). Also, push-ups and VO_2_max results were negatively correlated with %BF (*p* < 0.05). The strongest negative correlation was found between VO_2_max and %BF (*r* = −0.39).

WHR was negatively correlated with flexibility (*r* = −0.15; *p* < 0.05); however, the most significant correlations were found for the physical activity clusters (*Niche PA*, *Popular PA*, *Walking/running PA*). Again, *Sweets* were negatively correlated with weight categories and %BF (*r* = −0.11; *r* = −0.14; respectively; both at *p* < 0.05).

We computed correlation coefficients for participants according to their sex. The highest negative correlation in girls (see Table 4) was found between WHR and standing long jump (*r* =−0.31; *p* < 0.05). It was surprising that grip strength results were positively correlated with weight categories and %BF but negatively correlated with WHR (all of them at *p* < 0.05; *r* = 0.13, *r* = 0.27, *r* = −0.28; respectively).

We found similar correlations between grip strength and weight indices when analysing the boys’ data (Table 4). There were significant positive correlations between grip strength results and those of other weight categories (*r* = 0.20) and %BF (*r* = 0.12), whereas grip strength yielded a negative correlation with WHR (*r* = –0.20).

The rest of the physical fitness tests, except for flexibility, i.e., sit-ups, standing long jump, push-ups, and VO_2_max, were negatively correlated with the weight categories, %BF, and WHR. All of these correlations were statistically significant (*p* < 0.05). The strongest correlation was found between VO_2_max and %BF (*r* = −0.44).

Surprisingly again, rather low, but significant negative correlations were found between sweet dietary habits and weight categories and %BF.

### 3.3. Multinomial Logit Model

Given that Vo_2_max had the strongest correlation with weight indices in most of the age and sex subgroups, we decided to use it in further analysis. We applied a multinomial logit model, using BMI categories as a dependent variable (four levels: underweight, normal weight, overweight and obese) and Vo_2_max as a continuous (independent) variable. This was undertaken to assess the constant effect of Vo_2_max, as a predictor, on the likelihood of obesity. The group consisting of obese children served as the baseline. The model fit was tested with the Hosmer and Lemeshow test [41]. The pseudo-R^2^ = 0.88 confirmed the good fit of the model. The odds ratios are presented in Table 6. The VO_2_max increased by 13% in groups of overweight children, by 26% in normal weight and by 19% in underweight children while compared to the group of obese children.

## 4. Discussion

In this study, we examined the roles of diet, physical fitness, sedentary behavior and physical activity patterns in the prevalence of overweight and obesity among school children in the Lower Silesia region of Poland. The main finding was that VO_2_max, an index of cardiorespiratory fitness, was the most consistent correlate of %BF among the participants. This finding was observed in both gender and age groups, but sit-ups and standing long jump were strongly correlated with weight indices in 10–11-year-old children compared to other age groups. This finding is consistent with previous cross-sectional studies showing an inverse relationship between aerobic capacity (VO_2_max) and body fat in children [42,43]. Given that there is substantial literature on links between physical activity, aerobic capacity and obesity [44,45], the trend we found is not surprising.

Notably, WHR and BMI, other indices of adiposity were hardly correlated with physical fitness, physical activity, sedentary behavior and diets in the present study. Although there was a strong significant correlation between %BF and BMI, which is consistent with previous findings, our findings may just be a reflection that BMI and WHR are often not a good marker of adiposity compared to %BF. According to Ketel et al. [46], the percentage of body fat calculated from skinfold measurements may be a superior measure of fatness in the Caucasian population, whereas WHR may be not a reliable indicator of fat distribution [46], especially among children who have different body proportions compared to adults.

We found sit-ups and standing long jump as correlates of %BF and BMI, but trends were inconsistent across gender and age groups. Surprisingly, we observed contradictory correlations between weight indices and grip strength. For BMI categories and %BF, the correlation was positive, while for WHR and the grip strength test, a negative association was found. We could argue that this finding is related to disproportions of the children’s bodies, and as such, BMI and %BF, as highly correlated, indicate larger muscle mass rather than adiposity.

We did not notice steady correlations between the physical activity groups that the children were involved in and weight indices. The *Walking/running PA* was inversely correlated with all of the weight indices, but correlations were relatively weak and inconsistent across sex and age groups. Also, other physical activities correlated with one weight index but not with the others (e.g., *Niche PA* correlated with WHR and BMI whereas *Popular PA* only with WHR; see Table 4). Hence, these results help us to associate one single factor that could be used to determine overweight or obesity risk.

Surprisingly, but not consistently, the cluster that referred to *Sweets* correlated negatively with the BMI categories and %BF, suggesting that the more often you eat sweets, the lower BMI and less %BF you have. What is inconsistent is that this correlation was not found in 12-year-old children and in girls. It is also worth emphasizing that this correlation was rather weak, although significant in the whole examined population. We can speculate that children admitting having consumed *Sweets* more frequently than other food items but were at the same time more physically active, either in sport or at their domestic chores, which could explain our results.

Given that the correlation between dietary habits and weight indices also did not yield definite results, the only factor that consistently proved to be correlated with %BF in all analyses was Vo_2_max. The Vo_2_max increased in overweight, normal weight and underweight by 13, 26 and 19%, respectively, as compared to obese children. There is a strong relationship between physical activity and VO_2_max [47] and physical activity and obesity [5]. These relationships could also explain our results quite well.

It should be noted, however, that we did not find any particular type of physical activity that could be specifically associated with weight reduction. Very low correlations between the clustered physical activity types or sedentary behavior suggest that the process is more complex, and a set of different factors may be associated with normal weight rather than one behavioral factor, e.g., a specific type of physical activity or sedentary behavior. It may be hypothesized that children’s involvement in any type of physical activity increasing their aerobic capacity would reduce the prevalence of overweight and obesity. However, the physical activity may be the *inus* condition, i.e., insufficient but non-redundant parts of a condition which is itself unnecessary but sufficient for the occurrence of the effect, which is, in our case “a normal weight”. Perhaps not an aerobic activity but habitual physical activity, though usually not considered as aerobic exercise, is vital for weight reduction. Since physical activity involvement also changes (probably reduces) time spent on sedentary behavior and transportation pattern and, perhaps, affects diet, it may be speculated that physical activity itself, at least in the population that was described in this study, is the crucial factor. It is therefore fundamental to involve more children in physical activities in and out of school. In the first assessment on a Polish children’s physical activity level using the Active Healthy Kids Global Alliance grading system [48], the overall physical activity level, organized sport participation and sedentary behavior were graded D. This means that only 21–40% of children at the age of 9–17 years met the defined and recommended benchmark for these behavioral indicators. It seems that Polish children move too little, sit too much and consequently have somewhat limited opportunity to increase their aerobic capacity.

Some limitations of this study have to be acknowledged. Firstly, according to the cross-national survey OLAF [28] almost 20 percent of Polish children are overweight or obese, a tendency that was also noted in our sample that consisted of 13.1% and 4.06% of overweight obese children, respectively. However, our sample was not representative of the entire Polish population. Secondly, the method that we used in our study, i.e., self-reported assessments of diet and physical activity, regardless of their high reliability coefficients, should be interpreted cautiously. More objective measures could be applied (such as direct observations) since there is a possibility that children may have overestimated or underestimated either dietary intake or physical activity involvements. Thirdly, VO_2_max was estimated using a 20-m shuttle run test, which, although a reliable measure of aerobic capacity [47], is an indirect measurement of this attribute. The last limitation relates to the sampling method. Since we initially planned to build an SEM model, and therefore, we calculated the sample size considering the requirements for modeling, a study that will use a representative sample of the whole Polish school children population is needed.

The strength of this study to be acknowledged is that it is the first study that focuses exclusively on the relationships between diet, physical fitness, sedentary behavior and physical activity types and the prevalence of overweight and obesity among Polish school children, specifically in the Lower Silesia region. It is worth emphasizing that we focused on a behavioral perspective, i.e., on factors (e.g., type of physical activity, type of sedentary behavior) or health-related attributes (i.e., physical fitness) that may be associated with the prevalence of overweight and obesity. Country-specific interrogation and investigation of these factors is important as they may vary in different cultural and environmental contexts [49]. To our knowledge, this is the first study focusing on these aspects in the Polish population. We acknowledge that our study may be considered as preliminary, but it can serve as a very important foundation for understanding the influence of behavioral factors on development and management of overweight and obesity in Polish school children.

## 5. Conclusions

In this study, VO_2_max, an index of cardiorespiratory fitness, was the most consistent correlate of overweight and obesity among the participants. It could be concluded that education and intervention programs for increasing physical fitness (VO_2_max) through aerobic training should be recommended for physical education teachers, parents and children in order to reduce the rate of overweight and obese children in the Lower Silesia region of Poland. Appropriate monitoring, preventive and physical activity intervention programs for children should be designed. Such intervention activities could be carried out during physical education classes or at designated health centers.

### Perspective

In view of the results of our study, we could recommend the measurement of aerobic capacity at least every calendar year or in each grade as a pragmatic indicator of health risks associated with overweight and obesity in Polish school children. From our experience, indirect measures and field tests of aerobic capacity like the Cooper’s test or the 20-m multistage shuttle run test are practical and feasible for use by teachers to assess cardiorespiratory of children in Poland. Any values of VO_2_max below those commonly recommended for specific age and sex groups should be taken seriously as the children may be at risk of becoming overweight or obese. Based on our data a decrease of VO_2_max of about 25% could be regarded as a guide for weight reduction and obesity prevention among Polish children.

## Figures and Tables

**Table 1:** Descriptive statistics for anthropometric measurements.

Age Group (Years)	Sex	N	Height (m)		Body Mass (kg)		BMI (kg/m^2^)		Tricep Skinfold (mm)		Subscapular Skinfold (mm)		%BF		Waist Girth (cm)		Hip Girth (cm)		WHR	
			Mean	SD	Mean	SD	Mean	SD	Mean	SD	Mean	SD	Mean	SD	Mean	SD	Mean	SD	Mean	SD
10	girls	74	1.39	0.07	35.13	9.68	17.88	3.36	14.27	5.13	10.93	5.47	28.79	12.40	64.42	8.75	76.68	8.50	0.84	0.06
	boys	86	1.40	0.06	34.97	7.68	17.68	2.85	12.44	5.33	9.18	4.94	24.45	12.05	65.90	7.98	75.10	7.45	0.88	0.08
12	girls	71	1.50	0.10	40.13	9.46	17.70	2.86	13.57	4.61	9.04	3.87	25.65	9.62	66.54	8.56	81.01	9.94	0.83	0.09
	boys	66	1.50	0.08	42.92	10.3	18.81	3.17	13.32	5.38	9.78	6.05	26.24	12.89	69.90	9.69	81.39	7.97	0.86	0.06
14	girls	147	1.62	0.06	50.03	9.41	19.03	2.89	14.87	5.39	11.64	5.37	30.36	12.33	68.34	7.14	88.00	7.76	0.78	0.06
	boys	197	1.66	0.08	56.14	12.02	20.29	3.41	13.69	6.21	11.16	6.65	28.35	14.90	74.85	9.42	89.10	8.39	0.84	0.06

SD: standard deviation; BMI: body mass index ratio; % BF: percentage body fat; WHR: waist-to-hip ratio.

**Table 2:** Descriptive statistics for weight categories.

Age Group (Years)	Sex	Weight Categories							
		Under Weight		Normal Weight		Overweight		Obese	
		N	%	N	%	N	%	N	%
10	girls	8	10.8%	48	64.9%	11	14.9%	7	9.5%
	boys	8	9.3%	65	75.6%	9	10.5%	4	4.7%
12	girls	16	22.5%	51	71.8%	2	2.8%	2	2.8%
	boys	6	9.1%	45	68.2%	11	16.7%	4	6.1%
14	girls	25	17.0%	107	72.8%	14	9.5%	1	0.7%
	boys	11	5.6%	141	71.6%	37	18.8%	8	4.1%
Total (N)		74		457		84		26	

**Table 3:** Descriptive statistics of physical tests.

Age Group (years)	Sex	Flexibility (cm)		Sit-Ups (no./min)		Standing Long Jump (cm)		Push-Ups (no./min)		Grip Strength (kG)		VO_2_max (mL/kg/min^−1^)	
		Mean	SD	Mean	SD	Mean	SD	Mean	SD	Mean	SD	Mean	SD
10	girls	29.19	5.57	43.00	8.56	112.55	21.63	8.50	11.12	17.22	4.09	41.78	2.56
	boys	27.92	7.75	43.74	9.03	130.63	21.74	14.92	11.19	19.61	3.85	42.46	3.47
12	girls	28.92	7.77	44.03	7.78	134.44	23.66	7.32	9.59	21.56	5.00	41.09	3.65
	boys	25.45	7.55	44.61	9.80	147.70	23.15	19.00	14.09	23.29	4.52	43.20	4.91
14	girls	31.49	8.04	43.24	9.73	152.44	25.08	11.48	12.46	26.40	4.80	38.89	4.32
	boys	25.62	7.73	46.37	9.95	172.81	30.64	22.76	14.25	32.78	7.92	43.66	5.97

**Table 4:** Correlation coefficients calculated for all variables for all participants and for girls and boys separately. Bold and enlarged numbers indicate the statistical significance at *p* < 0.05.

	All Participants			Girls			Boys		
Variable	Weight Category (BMI)	%BF	WHR	Weight Category (BMI)	%BF	WHR	Weight Category (BMI)	%BF	WHR
Sit-ups	−0.16	−0.27	−0.12	−0.11	−0.25	−0.1	−0.23	−0.28	−0.22
Standing long jump	−0.13	−0.21	−0.14	−0.18	−0.14	−0.31	−0.2	−0.23	−0.24
Push-ups	−0.1	−0.27	−0.04	−0.05	−0.11	−0.19	−0.27	−0.36	−0.18
VO_2_max	−0.15	−0.39	0.05	−0.11	−0.28	0.02	−0.29	−0.44	−0.12
Grip strength	0.21	0.14	−0.11	0.13	0.27	−0.28	0.2	0.12	−0.2
Flexibility	0.02	−0.03	−0.16	0.03	−0.02	−0.16	0.08	−0.07	−0.02
Technical PA	−0.08	−0.02	0	−0.08	−0.02	−0.06	−0.07	−0.03	0.05
Niche PA	−0.11	−0.07	−0.08	0.01	−0.09	0.1	−0.11	−0.13	0.04
Popular PA	0.03	−0.03	0.19	−0.01	−0.05	0.13	−0.01	0.01	0.12
Walking/running PA	−0.14	−0.11	−0.11	−0.06	−0.05	−0.11	−0.19	−0.15	−0.07
Active	−0.01	0.04	−0.08	0.05	0.05	−0.01	−0.01	0.02	−0.01
Music	0.03	0.07	−0.11	0.02	0.06	−0.1	0.08	0.05	−0.04
TV/Computer	0.06	0.02	−0.1	−0.05	0.01	−0.15	0.11	0.05	−0.16
Every Day Diet	−0.07	−0.05	−0.04	−0.12	−0.08	0.05	−0.02	−0.04	−0.08
Meat and oil	0.01	−0.02	−0.02	0.04	0.06	−0.01	−0.05	−0.05	−0.11
Healthy	0	−0.02	0.04	−0.03	−0.01	0.03	0.02	−0.02	0.04
Sweets	−0.15	−0.16	−0.06	−0.18	−0.1	−0.04	−0.14	−0.19	−0.1
Fast Food	−0.06	−0.11	0.07	−0.09	−0.06	0.1	−0.07	−0.14	−0.02

**Table 5:** Correlation coefficients calculated for all variables for 10-, 12- and 14-year-old children. Bold and enlarged numbers indicate the statistical significance at *p* < 0.05.

	10 Years Old			12 Years Old			14 Years Old		
Variable	Weight Category (BMI)	%BF	WHR	Weight Category (BMI)	%BF	WHR	Weight Category (BMI)	%BF	WHR
Sit-ups	−0.22	−0.35	−0.06	−0.21		−0.17	−0.11	−0.23	−0.10
Standing long jump	−0.22	−0.37	−0.05	−0.03	−0.24	−0.03	−0.15	−0.32	0.04
Push-ups	−0.16	−0.28	0.02	−0.06	−0.28	0.09	−0.10	−0.32	−0.02
VO_2_max	−0.23	−0.34	−0.02	−0.30	−0.44	−0.14	−0.10	−0.39	0.11
Grip strength	0.26	0.21	0.03	0.34	0.23	−0.01	0.30	0.03	0.15
Flexibility	0.09	0.00	−0.08	−0.06	−0.24	−0.29	0.01	0.02	−0.15
Technical PA	−0.04	0.05	0.05	−0.09	−0.10	−0.02	−0.08	−0.05	0.05
Niche PA	0.04	0.06	−0.09	−0.26	−0.16	−0.13	−0.16	−0.05	−0.21
Popular PA	0.01	0.06	0.11	0.03	0.01	0.07	0.06	−0.03	0.22
Walking/running PA	−0.12	−0.17	−0.01	−0.20	−0.19	−0.08	−0.14	−0.05	−0.20
Active	0.08	0.15	−0.11	−0.13	−0.06	−0.14	−0.01	0.09	−0.17
Music	−0.03	−0.07	−0.01	0.02	0.06	0.00	0.08	0.08	−0.08
TV/Computer	0.05	0.03	−0.16	0.20	0.14	0.11	0.00	−0.08	−0.01
Every Day Diet	−0.11	−0.02	−0.05	−0.14	−0.11	−0.05	−0.02	−0.03	−0.08
Meat and oil	0.03	0.05	−0.06	−0.01	0.03	0.02	0.00	−0.06	−0.02
Healthy	−0.09	−0.06	0.04	0.02	0.05	0.02	0.04	0.00	−0.01
Sweets	−0.25	−0.25	−0.05	−0.08	−0.10	0.02	−0.11	−0.14	−0.08
Fast Food	−0.15	−0.15	−0.07	0.01	0.03	0.08	−0.06	−0.13	0.03

**Table 6:** Multinomial logit model results, where the independent variable was weight categories according to BMI and the continuous variable (predictor) was VO_2_max.

Weight Categories (BMI)	Effect	Estimate	Odds Ratio	Standard	Lower CL	Upper CL	*p*
					95.00%	95.00%	
Underweight	Intercept	−6.00	1.19	2.24	−10.39	−1.62	0.007
	VO_2_max	0.18		0.06	0.07	0.29	0.002
Normal weight	Intercept	−6.46	1.26	2.05	−10.48	−2.44	0.002
	VO_2_max	0.23		0.05	0.13	0.34	0.000
Overweight	Intercept	−3.46	1.13	2.20	−7.78	0.86	0.117
	VO_2_max	0.12		0.06	0.01	0.23	0.037

CL: the confidence level.

**Figure A1:**
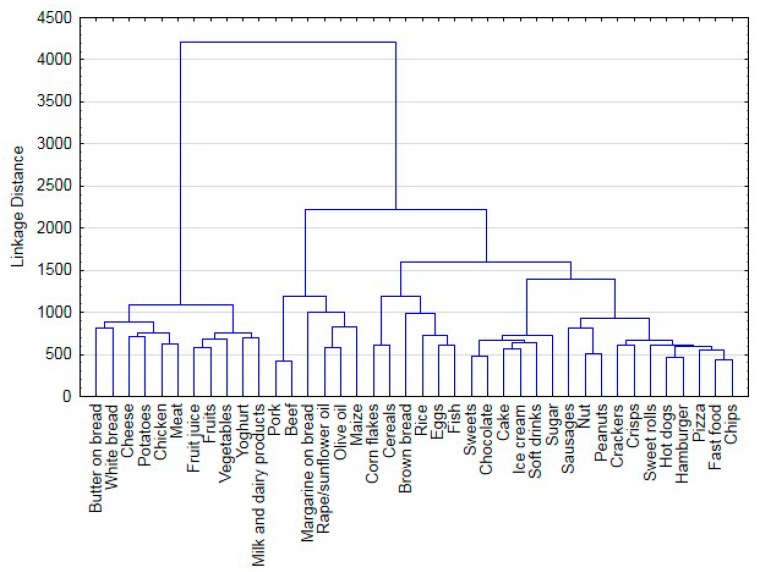
Tree diagram for cluster analysis of personal dietary assessment. Ward’s method was used. The Manhattan distances were used for measuring.

**Figure A2:**
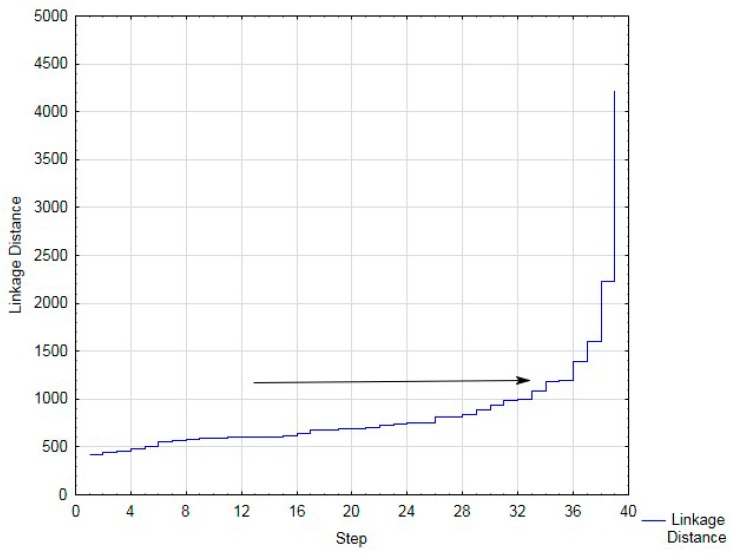
The plot of linkage distances across steps in personal dietary questionnaire cluster analysis. The arrow indicates the cut-off point and is equal to the distance of 1250.

**Figure A3:**
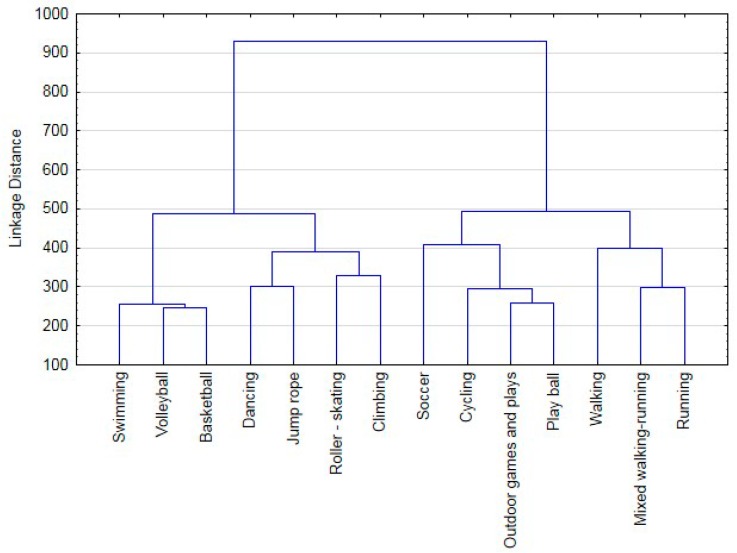
Tree diagram for cluster analysis of physical activities the children participated in. The cut-off point is at the distance of 450, clustering variables into four groups.

**Figure A4:**
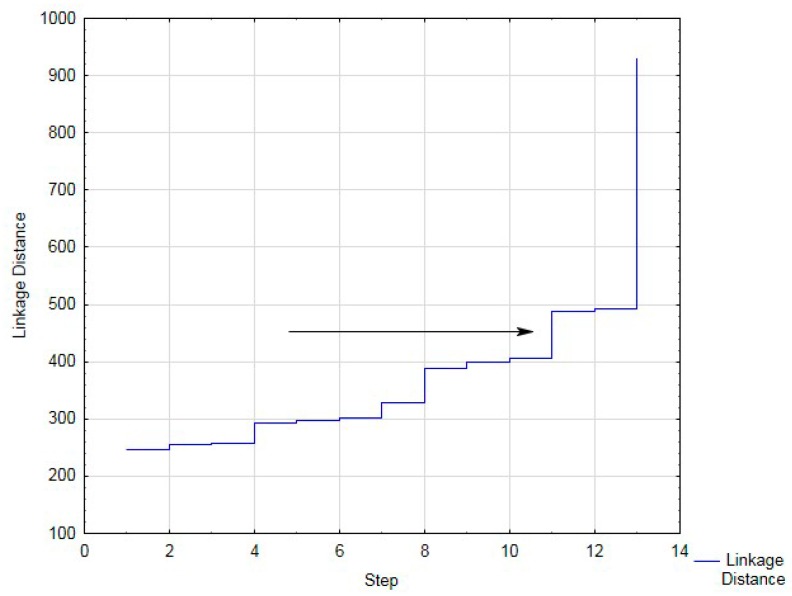
The plot of linkage distances across steps for cluster analysis of physical activities the children participated in. The arrow indicates the cut-off point and is equal to the distance of 450.

**Figure A5:**
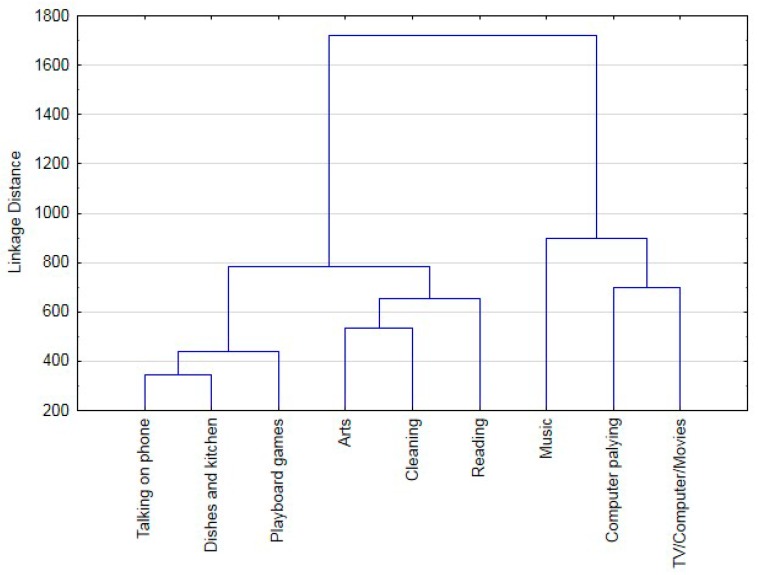
Tree diagram for cluster analysis performed for question referring to sedentary behaviors. Ward’s method was used. The Manhattan distances were used for measuring. The cut-off point is at the distance 850, clustering variables into four groups.

**Figure A6:**
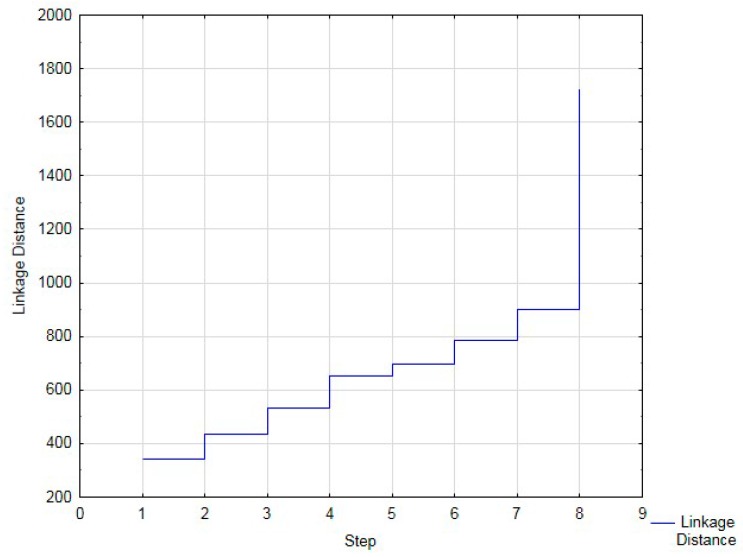
The plot of linkage distances across steps in cluster analysis performed for question referring to sedentary behaviors.

## Supplementary Materials

The following are available online: www.mdpi.com/1660-4601/14/6/664/s1, Table S1: Raw data.

## Appendix A

### Cluster Analysis Results

#### Personal Dietary Questionnaire Cluster Analysis

In order to examine the children’s dietary data, we performed a cluster analysis. As it can be noticed, some of the questions were broader than the others, e.g., in one question participants were asked how often they consume meat, while in the others they were asked about the most popular meat in Poland, i.e., beef, pork, and chicken. We assumed that older children could be able to recognize different kinds of meat, while the younger may not; however, in the cluster analysis, any discrepancies between the answers should be identified. If the answer for “Meat” in general and “Chicken”, Beef”, “Pork” did not belong to the same cluster, then it could be attributed to the children’s misunderstanding of the question. Similarly, “Fast food” is a general kind of food, one can even think of cold meat, and more specifically, foods like: “Hamburger’; “Pizza’, “Hot dog”, “Sausages”. Moreover, we assumed that listing the type of food that includes more specific types (such as meat) could be useful for those who could not find more specific food listed (e.g., lamb or ostrich are available in Poland but are not very popular).

In the cluster analysis, we used Ward’s method; city-block (Manhattan) distances as a distance measure (Figure A1).

We recognized five clusters. In the first one, the following food items were included: chips, fast food, pizza, hamburger, hot dogs, sweet rolls, crisps, crackers, peanuts, nuts, and sausages. We labeled this group as *Fast food* since most of the products could be characterized as fast food. The second cluster consisted of sugar, soft drinks, ice cream, cakes, chocolate, and sweets. Due to the fact that all of these products usually contain much sugar, we called them *Sweets*. In the third cluster, the following were included: fish, eggs, rice, brown bread cereals, and corn flakes. We categorized this cluster as *Healthy* since most of the products are advertised as “healthy food” and could be associated with health and fitness. The fourth cluster consisted of pork, beef, margarine on bread, olive oil, rape/sunflower oil, and maize. This cluster was called *Meat and oil*. As there can be a rationale for including oil and meat in the same category, there was a problem with maize since it is difficult to evince the association between maize and meat and oil. Perhaps, what the maize looks like (as a mash) raises such association in children. The last group consisted of different kinds of food: butter, white bread, cheese, potatoes, chicken, meat, fruit juices, fruits, vegetables, yogurt, milk and dairy products. Interestingly, all of the milk and dairy products were included in this category, i.e., cheese, yogurt, milk and dairy products. Also, fruits, fruit juices, and vegetables were included. The food items that were difficult to categorize in this cluster included: white bread, butter on bread, potatoes, chicken, and meat. We speculated that these products could likely have been used for the children’s last breakfast or lunch and therefore, easily remembered, were found in one category. We called it *Every day diet*. Values representing each observation in the clusters were computed as an arithmetic mean of the original values of the variables included in each cluster.

Our rationale for recognizing the aforementioned five clusters, apart from the already provided one, was supported by the graph of amalgamation schedule (Figure A2). In our opinion, the clear *plateau*, that may serve as a cut-off point for the tree diagram can be noticed at the distance of 1250.

#### Participation in Physical Activities Cluster Analysis

We performed cluster analysis for the physical activities that children could recall that they participated in on the previous day. We used Ward’s method; city-block (Manhattan) distances as a distance measure (Figure A3).

The plot of linkage distances suggested that the most rational cut-off point would be at the distance of 450 (Figure A4); however, the plateau was not as clear as in the previous cluster analysis. Therefore, we had four clusters, each including variables that can be rationally linked.

In the first cluster, three physical activities were included: swimming, basketball, and volleyball. The rationale for the cluster is that these activities require good technical skills and they usually are not self-learned. They require equipment, coaches, infrastructure, and in most cases they are organized in dedicated clubs. What is specific for these activities is that the children’s participation in them is usually initiated by parents. Therefore, we called this cluster *Technical PA*.

The second cluster consisted of four activities: dancing, jump-rope, roller-skating and climbing. These activities are not very popular among children in Poland. We called this cluster *Niche PA*. The low popularity of these activities may be reasoned in many ways. For example, the roller-skating requires roller skates that are relatively expensive and it may be one of the reasons why it is not very popular. Moreover, it requires a good infrastructure (roads, paths, skate parks, etc.) to ride on. Climbing also requires infrastructure that would allow children to climb. The jump-rope used to be a very popular activity years ago, but now it can be hardly noticed on playgrounds. Dancing is very specific, i.e., most of the young boys prefer other activities (e.g., [44]) and also in girls it is not the most popular activity. Usually, dancing is performed at clubs or homes. To attend dancing classes parents have to pay on monthly or course basis. On the other hand, homes may not be very popular, due to the limited space available, the lack of dancing partners, or social connotation (girls dancing with boys alone at home may have sexual connotations, especially among adolescents).

The four most popular activities, i.e., soccer, cycling, outdoor games and plays, and play ball were clustered in the third group. They are relatively costless, except for the bikes. Moreover, they do not require specifically arranged infrastructure, which means that you can play ball, soccer or cycle almost in all conditions. All of them can be performed with simply acquired motor skills. The cluster was called *Popular PA*. The fourth cluster consisted of three similar activities: running, walking and mixed walking/running. We called it *Walking/running PA*. Values representing each observation in the clusters were computed as an arithmetic mean of the original values of the clustered variables.

#### Sedentary Behaviors Cluster Analysis

Similarly, as in question one, we performed cluster analysis (Figure A5). However, the decision about the distance cut-off point was more difficult than in previous questions.

As it can be observed in Figure A6 there is no clear plateau that can serve as an optimal cut-off point.

Eventually, we decided to group our variables into three clusters (cut-off distance equal to 850). The one consisted of “watching TV/video/movies on computer” and “using a computer or playing video games”. The rationale for combining these two variables into one cluster was that both of them can be associated with passive time spending with the use of computer, TV, or video. We called this cluster *TV/Computer*. The second cluster consisted only of one element, i.e., listening and playing music. We called it *Music*. In the third cluster 6 different activities were included: reading books or magazines; playing board games; washing dishes or cleaning kitchen; talking on the phone, cleaning room/house; arts. This cluster consisted of quite different activities, some require physical activity (e.g., cleaning or washing dishes), whereas some do not (reading). However, all of them require self-activity either physical or mental from the children (e.g., doing arts or reading). Hence, we actually can differentiate this cluster from the *TV/Computer*. The third cluster was called *Active*. One would argue that also the variable “listening and playing music” could join this cluster; however, none of the methods for computing distances provided satisfactory results.
